# Coexisting Sigmoid Volvulus and Acute Meckel’s Diverticulitis Associated with a Fibrous Band: A Case Report

**DOI:** 10.3390/reports9040344

**Published:** 2026-10-12

**Authors:** Sergiu Balan, Claudiu Octavian Ungureanu, Razvan A. Stoica, Floris Cristian Stanculea, Niculae Iordache

**Affiliations:** General Surgery Department, Sf. Ioan Emergency Clinical Hospital, University of Medicine and Pharmacy “Carol Davila”, 020021 Bucharest, Romania; balan.sergiu1997@yahoo.com (S.B.);

**Keywords:** Meckel’s diverticulum, sigmoid volvulus, small bowel obstruction, omphalomesenteric band

## Abstract

**Background and Clinical Significance:** Meckel’s diverticulum is a remnant of the omphalomesenteric duct, arising from an incomplete obliteration of the embryonic vitelline duct, which connects the fetal gut to the yolk sac, typically regressing between the fifth and seventh weeks of gestation. There are many mechanisms for bowel obstruction arising from a Meckel’s diverticulum. Therefore, early diagnosis and treatment are the keys to prevent intestinal gangrene and perforation; **Case Presentation:** We report the management of sigmoid volvulus associated with acute Meckel’s diverticulitis and a fibrous band in an 85-year-old patient with multiple comorbidities and very high perioperative risk. Sigmoid volvulus was the mechanical cause of intestinal obstruction, whereas an inflamed Meckel’s diverticulum associated with a fibrous band extending toward the anterior abdominal wall was identified during surgical exploration. No twisting or mechanical obstruction of the distal ileum was observed. Histopathological examination subsequently confirmed acute Meckel’s diverticulitis. The unusual coexistence of these findings, together with the diagnostic and therapeutic challenges posed by the patient’s severe comorbidities, prompted us to report this case; **Conclusions:** Although no direct mechanical relationship between Meckel’s diverticulum and sigmoid torsion was demonstrated, their coexistence raises a hypothesis of a potential pathophysiological interaction; however, any such relationship remains hypothesis-generating only. Identification of one apparent cause of intestinal obstruction should not preclude systematic evaluation for concomitant pathology, particularly when the clinical, radiological, operative, and inflammatory findings are not fully explained by a single diagnosis.

## 1. Introduction and Clinical Significance

Meckel’s diverticulum (MD) is a remnant of the vitelline (omphalomesenteric) duct resulting from incomplete embryonic obliteration of the connection between the fetal gut and yolk sac [1,2]. It is the most common congenital anomaly of the gastrointestinal tract and is traditionally described by the ‘rule of 2s’: it occurs in approximately 2% of the population, is located within approximately 2 feet of the ileocecal valve, is about 2 inches long, may contain two common types of ectopic tissue (gastric and pancreatic), and is more likely to become symptomatic during the first 2 years of life [2]. Meckel’s diverticulum was first described by Fabricius Hildanus in 1598 and was subsequently named after Johann Friedrich Meckel the Younger, who established its embryological origin in 1809 [3]. It is located on the antimesenteric border of the ileum, at a reported mean distance of 52.4 cm from the ileocecal valve [4].

By definition, MD is a true diverticulum because its wall contains all layers of the small bowel [2,5]. Symptomatic disease occurs predominantly during the first three to four decades of life and has been reported up to four times more frequently in men [2,4,5]. Volvulus is a recognized but uncommon complication of MD [1,6]. Several mechanisms may lead to intestinal obstruction in patients with MD [6], including entrapment of small bowel around a fibrous band, intussusception, volvulus, and incarceration within a hernia sac [6,7]. Prompt diagnosis and appropriate surgical management are important to reduce the risk of intestinal ischemia, gangrene, and perforation [5,6]. Optimal management requires prompt recognition of Meckel’s diverticulum, followed by appropriate surgical decision-making and timely operative intervention [2,5,6].

Although intestinal obstruction is a well-recognized complication of Meckel’s diverticulum, the presence of Meckel’s diverticulum in a patient with bowel obstruction does not necessarily imply a direct mechanical relationship. Other independent causes of intestinal obstruction may coexist, particularly in older patients. Among the causes of acute large-bowel obstruction, sigmoid volvulus represents an important surgical condition characterized by torsion of a redundant sigmoid colon around its mesenteric axis [8,9]. Progressive torsion may compromise colonic perfusion, leading to ischemia, necrosis, and ultimately perforation if left untreated [8,9]. Although the anatomical predisposition to sigmoid volvulus is well recognized, the occurrence of an acute episode may also depend on additional precipitating factors capable of altering intestinal motility or intra-abdominal dynamics [10]. The coexistence of sigmoid volvulus and acute Meckel’s diverticulitis is therefore of particular clinical interest, as it raises the question of whether the two conditions represent independent simultaneous pathologies or whether an acute inflammatory process could contribute to volvulus formation in an anatomically susceptible sigmoid colon. However, in the absence of a demonstrated mechanical connection, such an interaction remains hypothetical.

## 2. Case Presentation

An 85-year-old man was admitted to our department with severe abdominal pain and a five-day history of absent intestinal transit. His medical history included NYHA class III congestive heart failure, chronic ischemic heart disease, chronic venous insufficiency of the lower limbs, calcified aortic atherosclerosis, first-degree atrioventricular block, vitamin B12 deficiency, and prostate adenocarcinoma previously treated with radiochemotherapy. His chronic medication included clopidogrel 75 mg daily. He had no history of previous abdominal surgery.

On admission, the patient appeared critically ill and required supplemental oxygen. Clinical examination revealed severe abdominal pain, mild peritoneal signs, marked abdominal distension with generalized tympanism, and visibly dilated bowel loops through the abdominal wall (Figure 1). Digital rectal examination revealed an empty rectal vault, without stool or evidence of rectal bleeding.

Laboratory investigations showed mild leukocytosis, thrombocytopenia (112 × 10^3^/μL), markedly elevated C-reactive protein (CRP; 323.4 mg/L), renal dysfunction (urea, 235 mg/dL; creatinine, 3.11 mg/dL), and hyperkalemia. Arterial blood gas analysis demonstrated mild acidemia (pH 7.30) with a decreased bicarbonate concentration (HCO_3_^−^, 18.7 mmol/L).

Abdominal computed tomography (CT) showed findings suggestive of sigmoid volvulus (Figure 2). Given the patient’s poor general condition and very high anesthetic and surgical risk, emergency colonoscopic decompression was initially selected as a less invasive therapeutic approach. Although the presence of mild peritoneal signs raised concern for bowel ischemia and would generally favor immediate surgical exploration, CT showed no evidence of intestinal ischemia or perforation. The associated Meckel’s diverticulum and fibrous band were not identified and were discovered during emergency laparotomy.

This finding, together with the exceptionally high perioperative risk, supported a carefully monitored attempt at endoscopic decompression. The colonoscope was advanced to approximately 40 cm from the anal verge, where marked luminal narrowing caused by extrinsic compression was identified. Given the inability to safely traverse the narrowed segment and the associated risk of perforation, endoscopic decompression was abandoned. This approach did not delay definitive treatment. Colonoscopy was promptly discontinued and emergency laparotomy was performed.

Intraoperatively, the sigmoid colon was markedly distended and ischemic (Figure 3), and a Hartmann procedure was performed. Further exploration revealed an inflamed Meckel’s diverticulum arising from the antimesenteric border of the distal ileum (Figure 4). The diverticulum was associated with a fibrous band extending from its apex toward the anterior abdominal wall, which, based on its anatomical configuration, was considered suggestive of a persistent omphalomesenteric remnant. Its presumed omphalomesenteric origin was based on the intraoperative anatomical findings and was not histologically confirmed. Segmental resection of Meckel’s diverticulum together with the involved ileal segment was performed, followed by primary intestinal anastomosis. The abdominal cavity was irrigated, hemostasis was secured, and a pelvic drain was placed before abdominal wall closure.

Gross examination of the resected colon showed a segment measuring 83.5 cm in length and up to 12.5 cm in maximal diameter. The serosal surface was brownish-purple with loss of normal sheen; the mucosa showed extensive ulceration, hemorrhage, and greenish surface deposits; and the bowel wall was flaccid, with focal necrotic thinning to approximately 1 mm. The second specimen consisted of a 6.7-cm ileal segment with a congested, edematous serosal surface and fecaloid intraluminal content. The diverticular wall was markedly thickened and showed gross features suggestive of acute diverticulitis.

Microscopic evaluation of the resected colon showed extensive mucosal ulceration covered by necrotic debris and hemorrhagic neutrophilic exudate, marked transmural vascular congestion and thrombosis, hemorrhagic extravasation, abundant acute suppurative granulocytic inflammatory infiltrates, and a moderate chronic lymphoplasmacytic inflammatory infiltrate.

The resected diverticular wall showed fibrino-hemorrhagic neutrophilic serositis, with areas of mucosal ulceration covered by a fibrino-hemorrhagic neutrophilic exudate. The lamina propria exhibited vascular congestion and hemorrhagic extravasation, associated with a moderate acute neutrophilic inflammatory infiltrate extending transmurally throughout the bowel wall. No ectopic gastric or pancreatic tissue was identified.

Although some of these histopathological findings may overlap with ischemic bowel injury, no torsion, strangulation, or mechanical obstruction was identified intraoperatively at the level of Meckel’s diverticulum or distal ileum. Therefore, the temporal relationship between the inflammatory and ischemic changes could not be determined from histology alone.

Exploration revealed an inflamed Meckel’s diverticulum associated with a fibrous band extending from the apex of Meckel’s diverticulum toward the anterior abdominal wall (Figure 4). Based on its anatomical configuration, the structure was considered suggestive of a persistent omphalomesenteric remnant; however, its embryological origin was not histologically confirmed.

Previously described mechanisms of Meckel-associated obstruction include rotation of adjacent ileal loops around a congenital fibrous attachment. This mechanism was not supported by the operative findings in our patient: no torsion or mechanical obstruction of the distal ileum was identified. The mechanical intestinal obstruction was instead caused by sigmoid volvulus, with marked colonic dilatation and ischemic changes.

The postoperative course was overall favorable. The patient required temporary vasopressor support and supplemental oxygen because of his pre-existing cardiopulmonary comorbidities. Bowel function subsequently recovered, and oral feeding was resumed. Recovery was prolonged by the patient’s significant comorbidities and a surgical wound infection requiring local wound care. Antibiotic therapy was discontinued on postoperative day 14, and the patient was discharged in stable condition on postoperative day 32. Systematic post-discharge follow-up was unavailable; therefore, subsequent wound healing, long-term stoma status, readmissions, further surgical interventions, and long-term survival could not be reliably assessed.

This case report was prepared and reported in accordance with the CARE guideline (Figure 5).

## 3. Discussion

During embryonic development, the vitelline (omphalomesenteric) duct connects the midgut lumen to the yolk sac. It normally undergoes obliteration during early gestation; incomplete involution may result in Meckel’s diverticulum. The diverticulum is a blind-ending pouch arising from the antimesenteric border of the ileum [2,5]. Approximately half of MDs may contain heterotopic mucosa, most commonly gastric mucosa, followed by pancreatic tissue [5]. The clinical manifestations of MD vary with age and complication type. Gastrointestinal bleeding is a common presentation in children, whereas intestinal obstruction is frequently reported among symptomatic adults [2,5]. Inflammation or perforation of MD may mimic other causes of acute abdomen, particularly acute appendicitis [2,5].

Several mechanisms may produce intestinal obstruction in patients with MD. These include entrapment of a bowel loop by a mesodiverticular band, intussusception, volvulus associated with a fibrous attachment, and incarceration within a Littre hernia [6]. In intussusception, inversion of the diverticulum may create a lead point that is propelled distally by peristalsis [11]. Secondary volvulus is rare but may compromise the vascular supply and produce a closed-loop obstruction [7,12]. Volvulus associated with MD is rare, particularly when a persistent congenital fibrous attachment is present [1,7,12]. Enterolith-related obstruction and perforation have also been reported [13]. These complications illustrate the heterogeneous mechanisms by which MD may present as an acute surgical abdomen.

In our patient, an inflamed Meckel’s diverticulum was identified on the antimesenteric border of the distal ileum. It showed marked wall thickening, edema, and hyperemia. A fibrous band extended from the diverticular apex toward the anterior abdominal wall. Importantly, the distal ileal loops were neither twisted around the band nor mechanically obstructed. The volvulus was located in the sigmoid colon rather than at the level of Meckel’s diverticulum or distal ileum. A distinctive feature of this case is the coexistence of sigmoid volvulus with histologically confirmed acute Meckel’s diverticulitis and an associated fibrous band. Previously reported cases of Meckel-associated volvulus generally describe a mesodiverticular or omphalomesenteric fibrous attachment acting as the anatomical axis of small-bowel rotation [1,7,12]. This mechanism was not demonstrated in the present case.

Although the fibrous band is considered a potential mechanism of small-bowel obstruction, operative exploration demonstrated neither twisting of the distal ileum around the band nor mechanical obstruction at this level. The mechanical cause of obstruction was sigmoid volvulus. Sigmoid volvulus results from torsion of a mobile sigmoid colon around its mesenteric axis, causing large-bowel obstruction and potentially vascular compromise. An elongated, redundant sigmoid colon with a relatively narrow mesenteric base is the principal anatomical substrate, although additional precipitating factors may contribute to torsion in susceptible patients [8,9,10,14,15].

Acute Meckel’s diverticulitis may produce substantial local and transmural inflammation. Recent reports describe phlegmon, purulent inflammation, serosal adhesions, and adherence of adjacent bowel loops or the diverticulum to the abdominal wall [16,17,18,19]. A recent adult case of perforated Meckel’s diverticulitis described an inflammatory mass involving the sigmoid colon together with the distal ileum, ileocecal region, and greater omentum [19]. These reports demonstrate that complicated Meckel’s diverticulitis can affect the local peritoneal environment beyond the diverticulum itself. In our case, however, no direct adhesion or mechanical connection was demonstrated between the inflamed Meckel’s diverticulum, the fibrous band, and the volvulated sigmoid colon. Neither Meckel’s diverticulitis nor the fibrous band can therefore be established as the direct cause of sigmoid torsion. The band should be regarded as an associated congenital finding rather than a demonstrated axis or fixation point for the sigmoid volvulus.

Nevertheless, the coexistence of histologically confirmed acute Meckel’s diverticulitis and sigmoid volvulus permits a cautious pathophysiological hypothesis. In an anatomically susceptible sigmoid colon, acute intra-abdominal inflammation may theoretically have contributed to torsion through transient changes in bowel motility, local peritoneal inflammation, or altered spatial relationships between mobile intestinal segments [10,16,17,18,19]. A direct causal relationship has not been established, and this proposed interaction should therefore remain explicitly hypothesis-generating.

Histopathological examination confirmed an active inflammatory process in Meckel’s diverticulum. However, mucosal ulceration, vascular congestion, thrombosis, hemorrhage, neutrophilic inflammation, and serositis may overlap with ischemic bowel injury. Accordingly, the histological findings do not establish the temporal sequence between inflammation and ischemic change.

The main strength of this report is the documentation of an unusual coexistence of sigmoid volvulus, histologically confirmed acute Meckel’s diverticulitis, and an associated fibrous band in an elderly patient with no previous abdominal surgery. The combination of clinical, radiological, endoscopic, intraoperative, and histopathological findings allowed reconstruction of the diagnostic and therapeutic course and clarified that the mechanical obstruction resulted from sigmoid volvulus rather than small-bowel torsion around the band.

The principal limitation is the inability to establish a causal relationship between Meckel’s diverticulitis and sigmoid volvulus. Although acute local inflammation may theoretically have contributed to sigmoid torsion in an anatomically susceptible colon, no direct adhesion, fixation point, or mechanical connection between the inflamed Meckel’s diverticulum, the fibrous band, and the sigmoid colon was demonstrated intraoperatively. In addition, Meckel’s diverticulum and fibrous band were not clearly identified on preoperative CT, limiting radiological correlation with the operative findings. As a single case report, this observation cannot determine whether the coexistence of these conditions reflects a pathophysiologically relevant interaction or a coincidental association. The proposed inflammatory contribution should therefore be regarded as hypothesis-generating. The absence of documented long-term follow-up is an additional limitation, preventing assessment of subsequent wound healing, stoma-related outcomes, readmissions, further surgery, and long-term survival.

From a clinical perspective, this case highlights the importance of considering the coexistence of more than one intra-abdominal pathology in elderly patients presenting with acute intestinal obstruction. Although sigmoid volvulus represented the actual mechanical cause of obstruction in our patient, acute Meckel’s diverticulitis was identified as a concomitant inflammatory condition and confirmed histopathologically. Importantly, the presence of a presumed omphalomesenteric band should not, in itself, be interpreted as evidence of band-related small bowel obstruction or volvulus when no ileal transition point, entrapment, or mesenteric torsion is demonstrated. In patients with sigmoid volvulus accompanied by an inflammatory response that appears disproportionate to the radiological findings, particularly when CT does not demonstrate bowel ischemia or perforation, an additional intra-abdominal inflammatory process should be considered. Careful evaluation of the distal ileum for an inflamed Meckel’s diverticulum may therefore be warranted, even when the primary obstructive pathology is located in the colon.

## 4. Conclusions

Contemporary reports demonstrate that complicated Meckel’s diverticulitis may produce extensive local inflammation, serosal adhesions, and involvement of adjacent intra-abdominal structures, including the sigmoid colon. This case illustrates the unusual coexistence of sigmoid volvulus, acute Meckel’s diverticulitis, and a persistent omphalomesenteric band. Although no direct mechanical relationship between Meckel’s diverticulum and sigmoid torsion was demonstrated, the acute inflammatory process may have represented a contributing or precipitating event in a predisposed sigmoid colon; however, this potential association remains speculative and should be regarded as hypothesis-generating rather than evidence of causality.

Identifying one apparent cause of intestinal obstruction should not preclude systematic exploration for concomitant pathology, particularly when clinical, radiological, operative, and inflammatory findings are not fully explained by a single diagnosis. Further reports are required to determine whether this association is incidental or reflects a clinically relevant pathophysiological interaction.

## Figures and Tables

**Figure 1:**
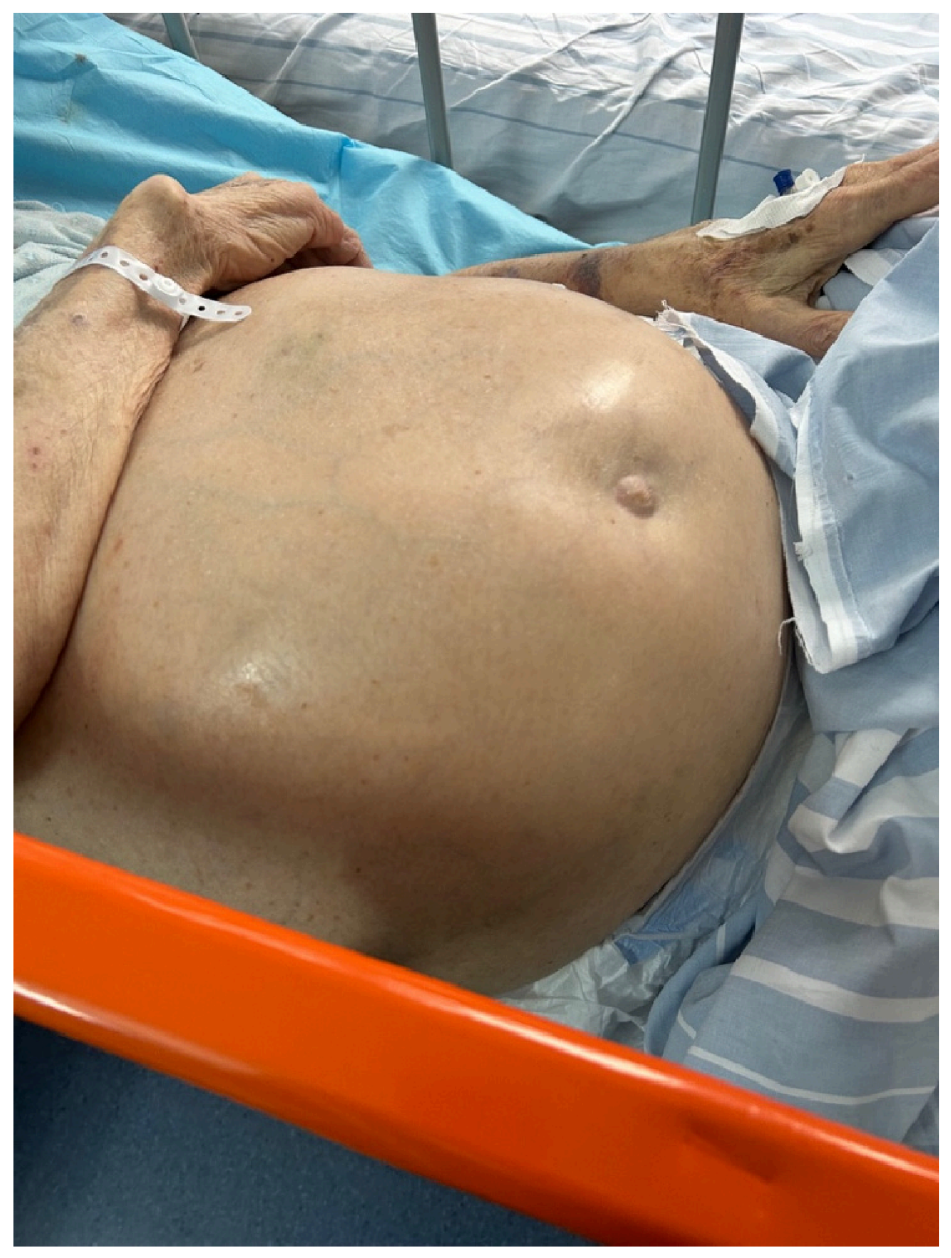
Clinical appearance at admission. Marked abdominal distension and visibly dilated bowel loops through the abdominal wall.

**Figure 2:**
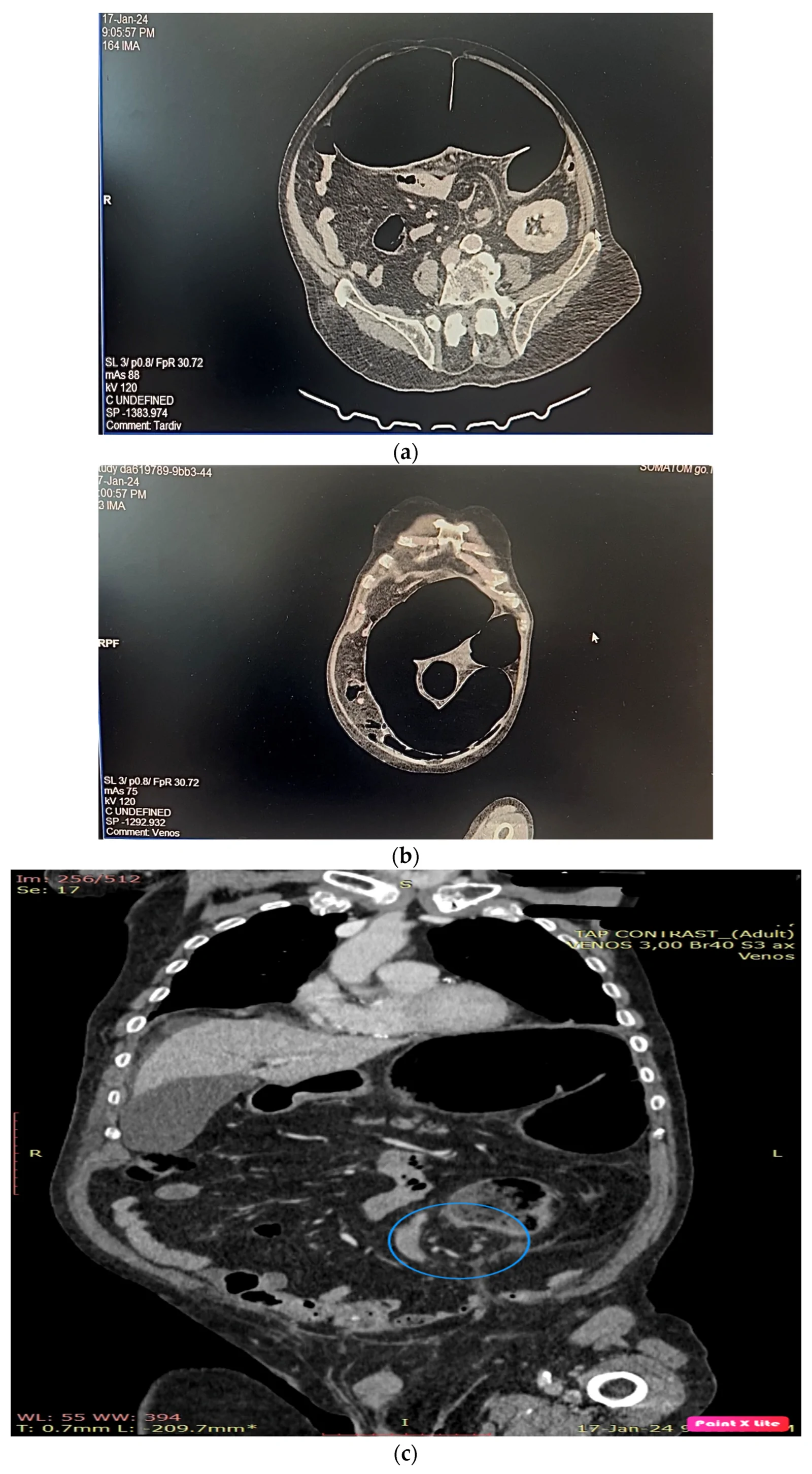

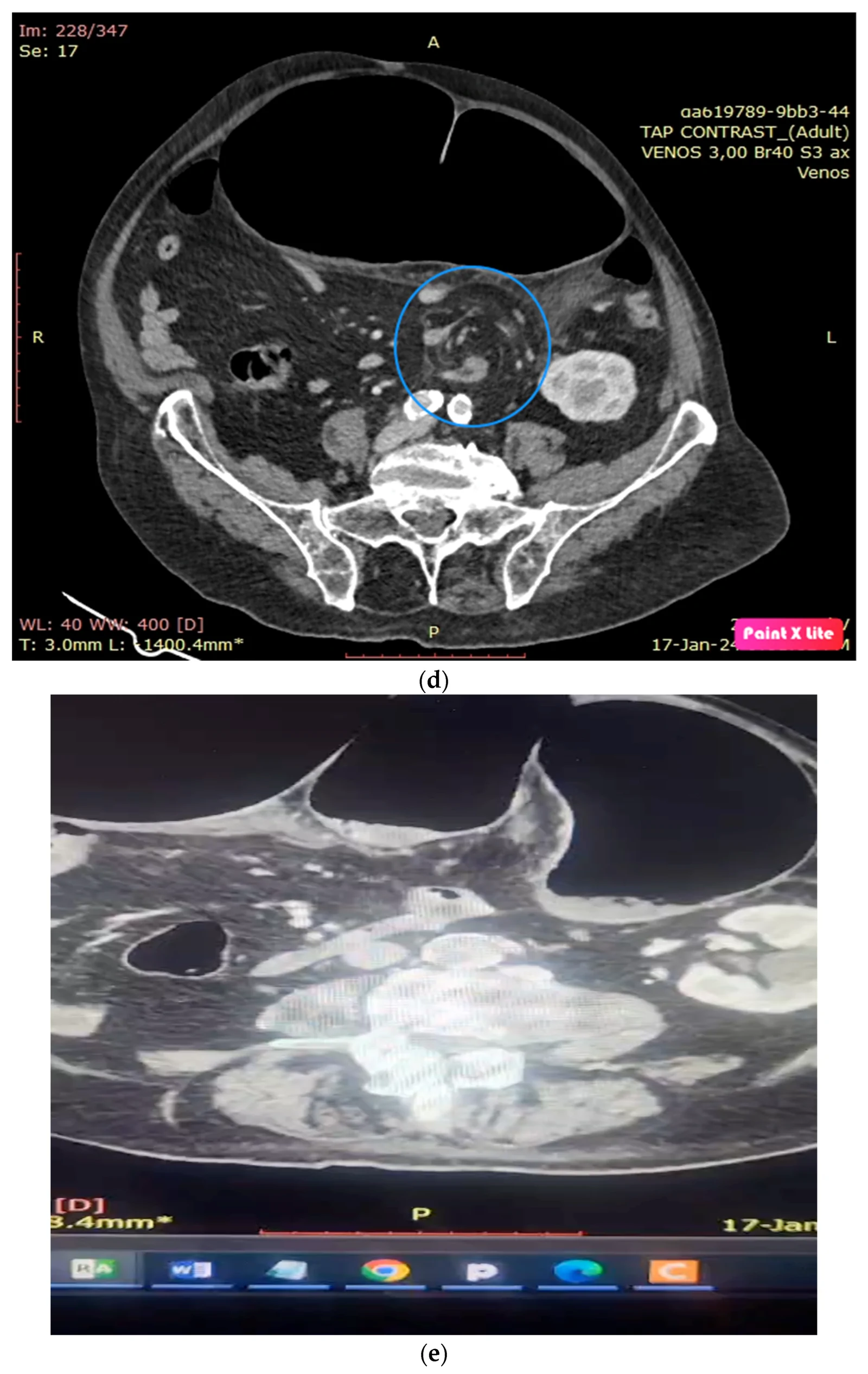
Preoperative contrast-enhanced abdominal CT. (**a**,**b**) Axial CT images demonstrating marked colonic distension in the setting of sigmoid volvulus. (**c**) Coronal CT reconstruction showing the twisted sigmoid mesentery, with the area of torsion highlighted. (**d**) Axial CT image demonstrating the whirl sign, with twisting of the mesenteric vessels and surrounding mesenteric tissue highlighted. (**e**) Additional axial CT image showing the mesenteric twisting associated with sigmoid volvulus.

**Figure 3:**
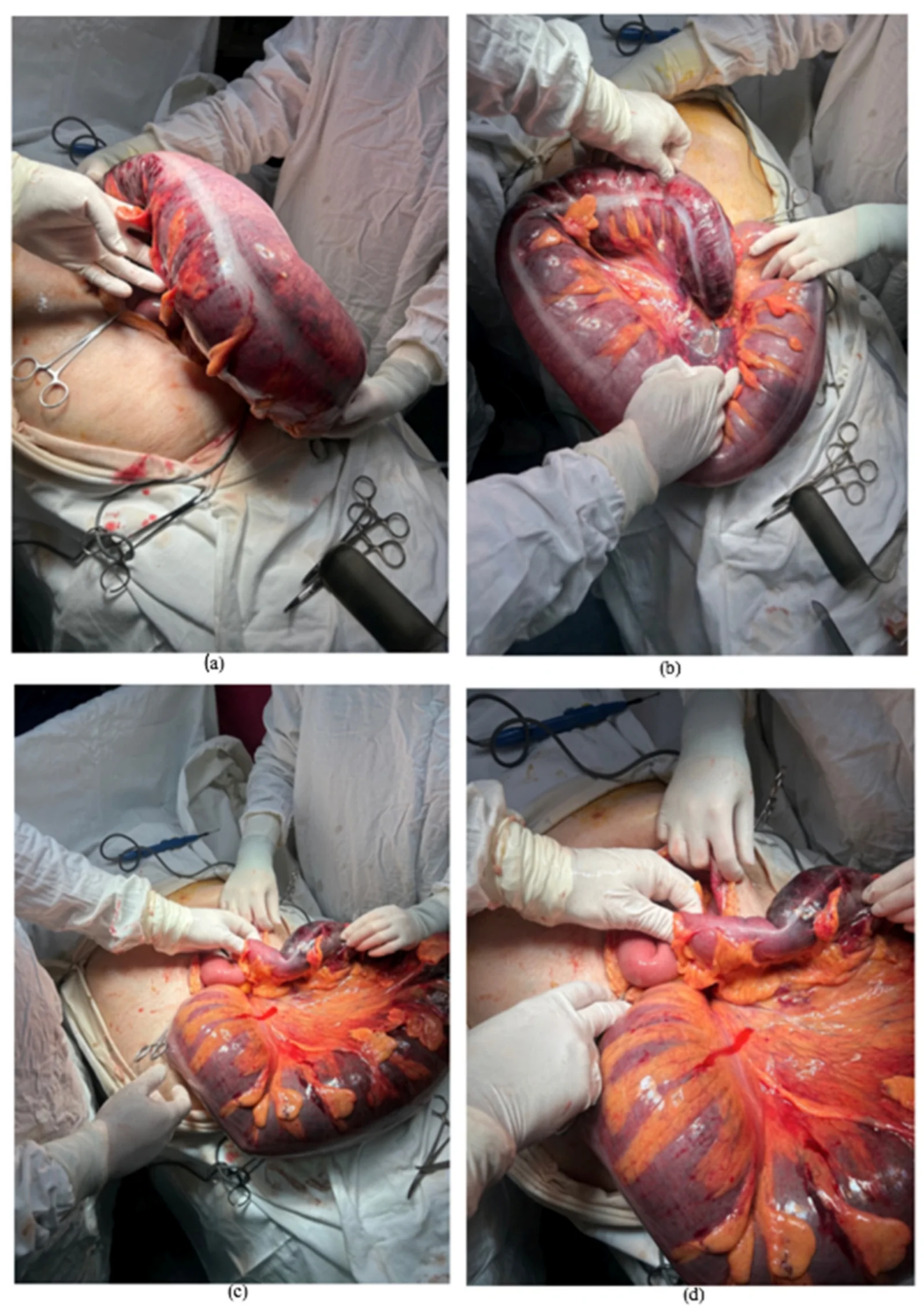
Intraoperative appearance of colonic volvulus. (**a**–**d**) Marked bowel distension, mesenteric torsion, venous congestion, and dark-purple discoloration of the affected bowel are visible, consistent with compromised intestinal perfusion.

**Figure 4:**
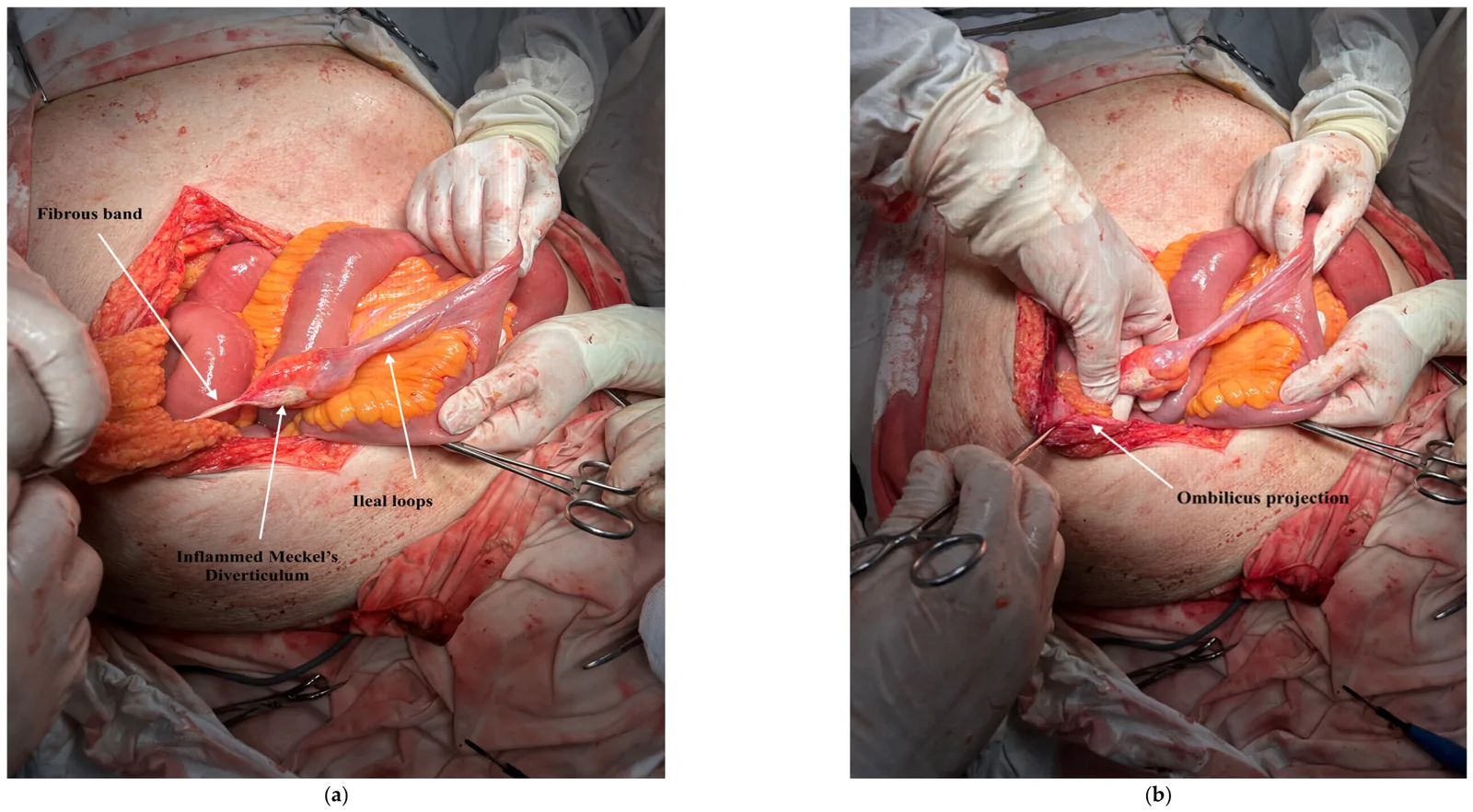
Intraoperative appearance of Meckel’s diverticulum and associated fibrous band. (**a**) Inflamed Meckel’s diverticulum arising from the antimesenteric border of the distal ileum, with the associated fibrous band extending from the diverticular apex. (**b**) Fibrous band extending from the apex of Meckel’s diverticulum toward the anterior abdominal wall in the direction of the umbilical region.

**Figure 5:**
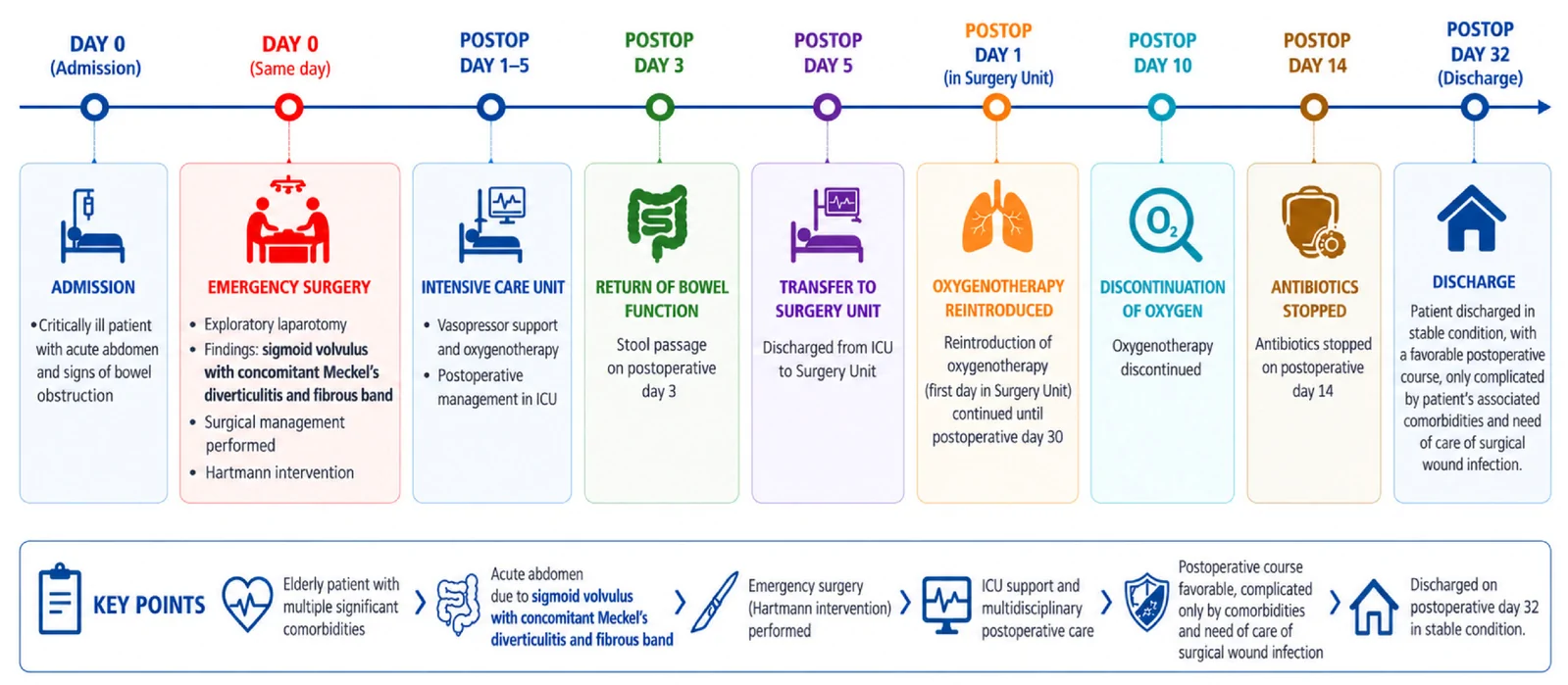
Timeline of the patient’s clinical course from admission to discharge.

## Data Availability

The data presented in this study are available on request from the corresponding author. The data are not publicly available due to patient privacy and confidentiality restrictions.

## Abbreviations

The following abbreviations are used in this manuscript:

MD	Meckel’s diverticulum
SBO	Small bowel obstruction
CT	Computed tomography
CRP	C-reactive protein
